# Ultramicronized *N*-Palmitoylethanolamine Supplementation for Long-Lasting, Low-Dosed Morphine Antinociception

**DOI:** 10.3389/fphar.2018.00473

**Published:** 2018-06-01

**Authors:** Lorenzo Di Cesare Mannelli, Laura Micheli, Elena Lucarini, Carla Ghelardini

**Affiliations:** Department of Neuroscience, Psychology, Drug Research and Child Health–Neurofarba–Pharmacology and Toxicology Section, University of Florence, Florence, Italy

**Keywords:** pain therapy, opioid, abuse, tolerance, microglia, astrocyte, PEA

## Abstract

The facilitation of opioid medication is eliciting a nemetic problem since increasing overdose deaths involve prescription of opioid pain relievers. Chronic painful diseases require higher doses of opioids, progressively with the development of tolerance to the antinociceptive effect. Novel strategies for the maintenance of low dosed opioid effectiveness are necessary to relieve pain and decrease abuse, overdose, and side effects. *N*-Palmitoylethanolamine (PEA) is an endogenous compound able to preserve the homeostasis of the nervous system and to delay the development of morphine tolerance. In the present study, a preemptive and continuative treatment with ultramicronized PEA (30 mg/kg, daily, *per os*) enhanced the acute antinociceptive efficacy of morphine (10 mg/kg subcutaneously) in rats and prolonged the responsiveness to the natural opioid. Moreover, PEA-treated animals had a more rapid recovery from tolerance. Four opioid free days were enough to regain sensitivity to morphine whereas control animals needed 31 days for full recovery of tolerance. Characteristically, PEA acquired *per se* antinociceptive properties in tolerant animals, suggesting the possibility of an integrated morphine/PEA treatment protocol. To maintain a significant analgesia, morphine dose had to be increased from 5 up to 100 mg/kg over 17 days of daily treatment. The same pain threshold increase was achieved in animals using preemptive PEA (30 mg/kg, daily) joined to a combinatorial acute treatment with morphine (5–20 mg/kg s.c.) and PEA (30–120 mg/kg, p.o.). Representatively, on day 17, the magnitude of analgesia induced by 100 mg/kg morphine was obtained by combining 13 mg/kg of morphine with 120 mg/kg of PEA. PEA strengthens the efficacy and potency of morphine analgesia, allowing prolonged and effective pain relief with low doses. PEA is suggested in association with morphine for chronic pain therapies distinguished by low risk of side effects.

## Introduction

The World Health Organization (WHO) defined the access to pain treatment a human right (2009). In particular, the WHO’s recognition of the absolute necessity of opioid analgesics has reflected the consensus among health experts for decades. Various other international bodies, such as the United Nations Economic and Social Council and the World Health Assembly, have also called on countries to ensure an adequate availability of opioid analgesics (Lohman et al., 2010).

Today, this indication has been transposed into national law in several countries increasing opioid prescriptions and quality of life related to pain relief.

On the other hand, a new phenomenon has arisen. Countries where the highest percentages of total world consumption of morphine occurs (North America and Europe) are experiencing problems related to the facilitation of opioid prescription as abuse, overdose, and dramatic side effects (Chou et al., 2015; Rudd et al., 2016a,b; Manchikanti et al., 2017). Persistent pain conditions, needing chronic therapies, amplify the major adverse consequences of opioids. The development of tolerance requires increasing dosages to obtain the same analgesic effect but generally enhances the severity of side effects as physical dependence, addiction, fatigue, cognitive dysfunction, dry mouth, sweating, and weight gain (Jonsson et al., 2010).

Improvement of opioid prescription guidelines, as recently suggested by a panel of experts, can offer a responsible and safe approach to this medical and social problem (Manchikanti et al., 2017). Moreover, the possible use of non-opioid drugs such as NSAIDs, anticonvulsants, or antidepressants should be considered for single cases verifying effectiveness and side effects. Nevertheless, the possibility of directly intervening in the mechanisms of the vicious cycle of tolerance development – dose increase – side effects and abuse remains an attractive approach.

The natural fatty-acid ethanolamide *N*-Palmitoylethanolamine (PEA), the endogenous amide between palmitic acid and ethanolamine, was recently identified as an endogenous molecule that intervenes with nervous alterations that lead to the lack of morphine antinociceptive effects (Di Cesare Mannelli et al., 2015a). In rats, PEA delayed the onset of morphine analgesic tolerance by preventing the activation of glial cells in the central nervous system, major players in the complex phenomenon of tolerance (Mika, 2008; Mika et al., 2009). Moreover, PEA protects nervous tissue in neuropathic conditions (Di Cesare Mannelli et al., 2013a), prevents neurotoxicity and neurodegeneration (Lambert et al., 2001; D’Agostino et al., 2012; Esposito et al., 2012), and inhibits peripheral inflammation and mast cell degranulation (Mazzari et al., 1996; Skaper et al., 2013). PEA decreases hyperalgesia without altering the normal pain threshold (Luongo et al., 2013; Di Cesare Mannelli et al., 2015b).

Considering the theoretical bases for a possible application of PEA in opioid-based therapies, we aim to define a preclinical protocol for PEA supplementation of morphine repeated treatment, focusing on the maintenance of a constantly high pain threshold over time with low opioid dosage.

## Materials and Methods

### Animals

For all the experiments described below, male Sprague-Dawley rats (body weight 200–250 g; Harlan, Varese, Italy) were used. Animals were housed in CeSAL (Centro Stabulazione Animali da Laboratorio, University of Florence) and used at least 1 week after their arrival. Four rats were housed per cage (size 26 cm × 41 cm) kept at 23 ± 1°C with a 12 h light/dark cycle (light at 7 a.m.), and were fed a standard laboratory diet and tap water *ad libitum*. All animal manipulations were carried out according to the Directive 2010/63/EU of the European parliament and of the European Union council (22 September, 2010) on the protection of animals used for scientific purposes. The ethical policy of the University of Florence complies with the Guide for the Care and Use of Laboratory Animals of the United States National Institutes of Health (NIH Publication No. 85-23, revised 1996; University of Florence Assurance No. A5278-01). Formal approval to conduct the experiments described was obtained from the Animal Subjects Review Board of the University of Florence. Experiments involving animals have been reported according to ARRIVE guidelines (McGrath and Lilley, 2015). All efforts were made to minimize animal suffering and to reduce the number of animals used.

### Treatments

**FIGURE 1:**
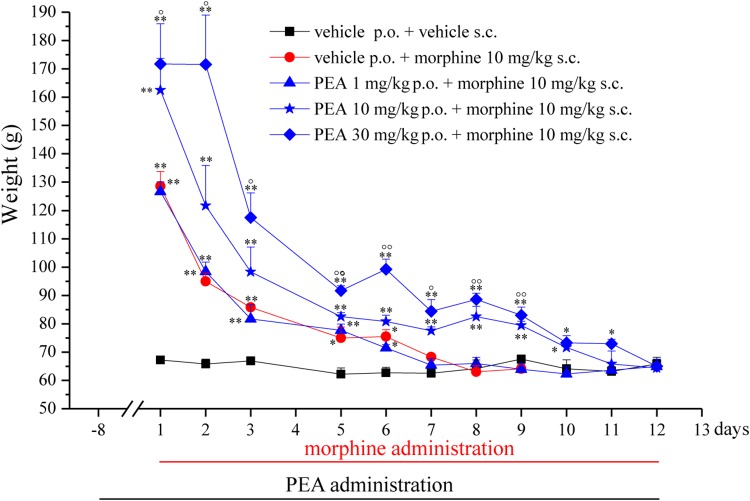
Effect of repeated administrations of PEA on the onset of morphine tolerance. PEA (1–30 mg/kg), or vehicle, was administered p.o. daily (in the evening) for the duration of the experiment starting on day -8. On day 1, daily, acute morphine treatment (10 mg/kg s.c.) began. Pain threshold measurements (Paw pressure test) were performed in the morning, 16 h after PEA administration and 30 min after morphine injection. Data are expressed as mean ± SEM of values from 12 rats analyzed in two different experimental sets. ^∗^*P* < 0.05 and ^∗∗^*P* < 0.01 vs. vehicle + vehicle; °*P* < 0.05 and ^∘∘^*P* < 0.01 vs. vehicle + morphine.

**FIGURE 2:**
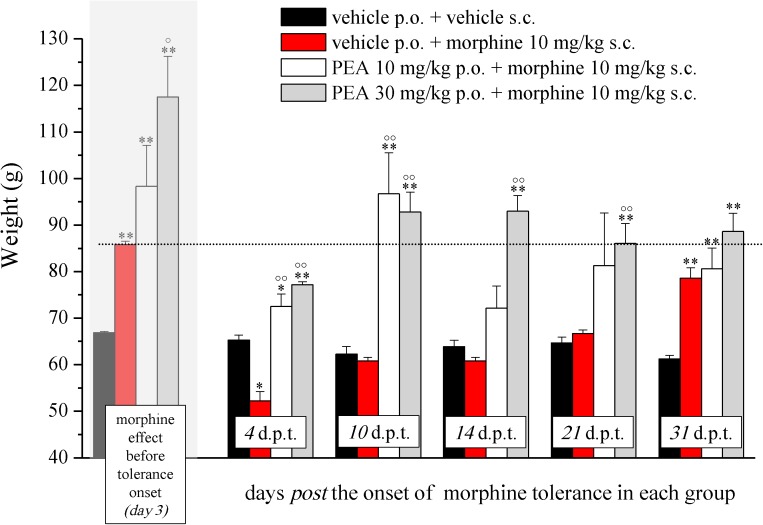
Effect of PEA on recovery from morphine tolerance. PEA (10 and 30 mg/kg), or vehicle, was administered p.o. daily (in the evening) for the duration of the experiment starting on day -8. Beginning on day 1, each group was treated daily with morphine (10 mg/kg s.c.). Representatively, the effect obtained on day 3 is shown (gray highlighted bars), the dashed line allows the comparison with the effects reached in the following days. Morphine treatment was continued till complete loss of antinociceptive response. The restoration of morphine efficacy was tested 4, 10, 14, 21, and 31 days *post* morphine tolerance onset (d.p.t.) in each group, respectively. Pain threshold measurements (Paw pressure test) were performed in the morning, 16 h after PEA administration and 30 min after morphine injection. Data are expressed as mean ± SEM of values from 12 rats analyzed in two different experimental sets. ^∗^*P* < 0.05 and ^∗∗^*P* < 0.01 vs. vehicle + vehicle; °*P* < 0.05 and ^∘∘^*P* < 0.01 vs. vehicle + morphine.

**FIGURE 3:**
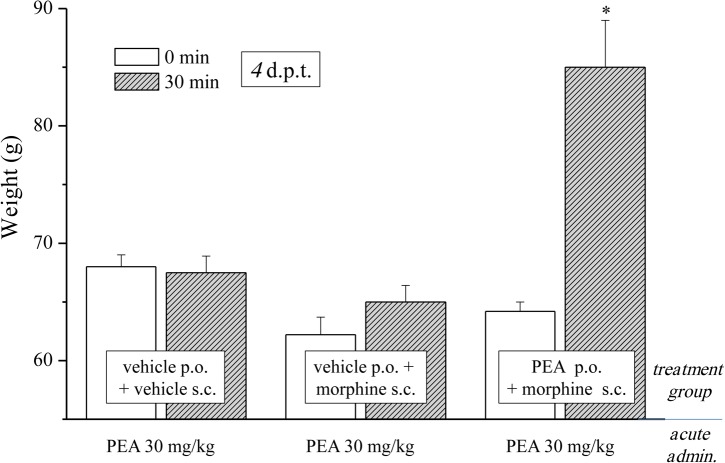
Effect of acute administration of PEA in morphine-tolerant animals. Rats were treated with vehicle or PEA (30 mg/kg) p.o. daily (in the evening) for the duration of the experiment starting on day -8. Morphine (10 mg/kg s.c.) was injected from day 1 till complete loss of antinociceptive response. When tolerance was well-established (4 d.p.t.), pain threshold (Paw pressure test) was measured in the morning before (0 min) and 30 min after an acute treatment with PEA (30 mg/kg, p.o.). Data are expressed as mean ± SEM of values from 12 rats analyzed in two different experimental sets. ^∗^*P* < 0.05 and ^∗∗^*P* < 0.01 vs. vehicle + vehicle, 30 min.

In the first phase experiments (**Figures 1–3**), ultramicronized PEA (Epitech Group, Padova, Italy – 30 mg/kg suspended in 1% carboxymethylcellulose) or vehicle were administered p.o. daily (in the evening) from day -8 to day 0 and from day 1 to the end of the experiment. Starting on day 1, up to the development of tolerance, acute treatments with morphine (S.A.L.A.R.S., Como, Italy – 10 mg/kg solubilized in 0.9% NaCl s.c.) were performed in the morning, 16 h after PEA. Measurements of pain threshold (Paw pressure test) were performed before (0 min) and 30 min after morphine injection. Tolerance was considered established the first day of complete loss of morphine antinociceptive effect (day 7, for vehicle + morphine and 1 mg/kg PEA + morphine groups; day 12, for 10 and 30 mg/kg PEA + morphine groups). Restoration of morphine efficacy was tested 4, 10, 14, 21, and 31 days *post* onset of morphine tolerance (d.p.t.) separately in each group. Also during this period, preemptive PEA treatment was performed daily in the evening (10 or 30 mg/kg), and Paw pressure test was performed in the morning 16 h after PEA administration and 30 min after morphine injection (10 mg/kg s.c.). The antinociceptive effect of acute PEA (**Figure 3**) was evaluated in morphine tolerant rats 4 d.p.t.

**FIGURE 4:**
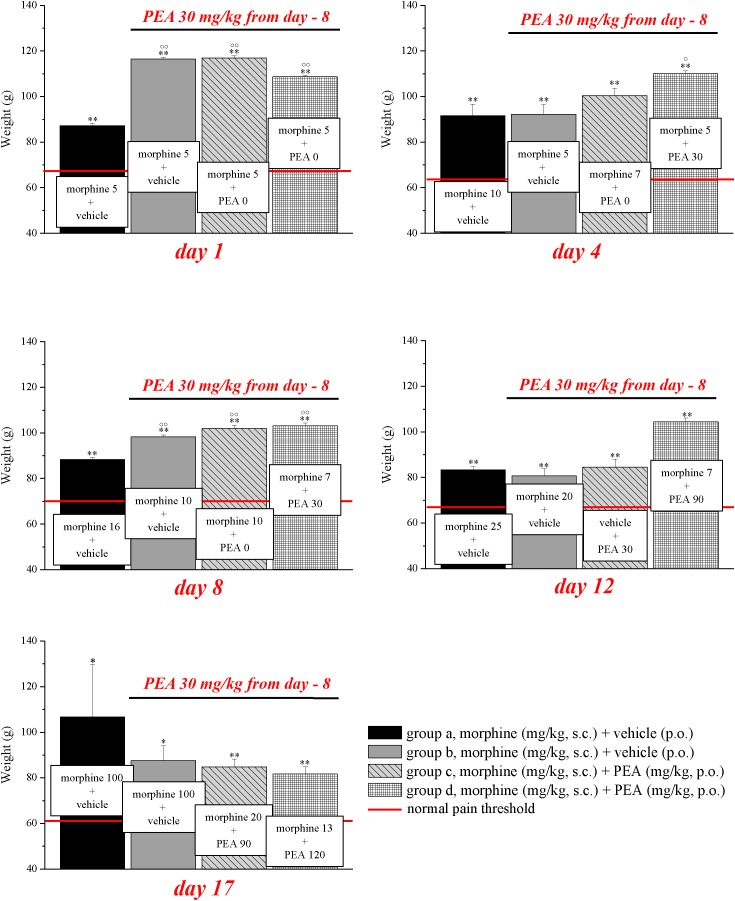
Antinociceptive effects of different combinations of morphine and PEA. Rats were treated with vehicle (group a) or PEA (30 mg/kg; groups b, c, and d) p.o. daily (in the evening) for the duration of the experiment starting on day -8. To maintain a significant increase of pain threshold (90 ± 10 g) vs. baseline (control; indicated with the red line), beginning on day 1, increasing daily doses of morphine (5–100 mg/kg) were injected s.c. to groups a and b. Different combinations of morphine (5–20 mg/kg, s.c.) and PEA (30–120 mg/kg, p.o.) were administered to groups c and d. Measurements were performed every day, in the morning, 30 min after morphine and/or PEA acute administration. Representative results obtained on days 1, 4, 8, 12, and 17 are shown. Data are expressed as mean ± SEM of values from 12 rats analyzed in two different experimental sets. ^∗^*P* < 0.05 and ^∗∗^*P* < 0.01 vs. the normal pain threshold (control, vehicle + vehicle + vehicle); °*P* < 0.05 and ^∘∘^*P* < 0.01 vs. group a.

**FIGURE 5:**
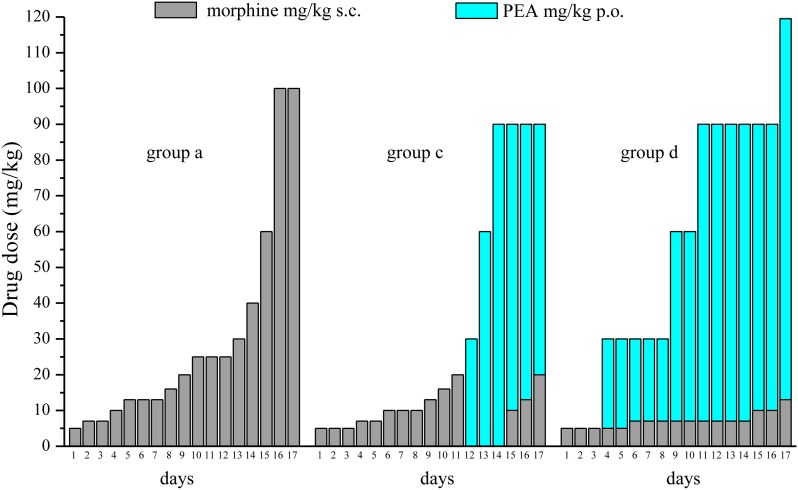
Comparison among equi-analgesic dosages of morphine/PEA combinations and morphine. Rats were treated with vehicle (group a) or PEA (30 mg/kg; groups c and d) p.o. daily (in the evening) for the duration of the experiment starting on day -8. To maintain a significant increase of pain threshold (90 ± 10 g), beginning on day 1, increasing daily doses of morphine (5–100 mg/kg) were injected s.c. to group a. Different combinations of morphine (5–20 mg/kg, s.c.) and PEA (30–120 mg/kg, p.o.) were administered to groups c and d. The dosages in mg/kg necessary to maintain the required antinociceptive effect every day (Paw pressure test; days 1–17) are reported. Measurements were performed in the morning, 30 min after morphine and/or PEA acute administration. Each group consists of 12 rats analyzed in two different experimental sets.

Experiments carried out in the second phase (**Figures 4, 5**), were performed on different groups treated, respectively, with vehicle (group a) or PEA (30 mg/kg; groups b, c, and d) p.o. daily (in the evening) for the duration of the experiment starting on day -8. To maintain a significant increase of pain threshold (90 ± 10 g) vs. baseline (control), beginning on day 1, daily increasing doses of morphine (5–100 mg/kg) were injected s.c. to groups a and b. Different combinations of morphine (5–20 mg/kg, s.c.) and PEA (30–120 mg/kg, p.o.) were administered to groups c and d. Control groups were pre- and acutely treated with PEA (group e, PEA + vehicle + PEA) or vehicle (group d, vehicle + vehicle + vehicle) without morphine injections. Pain measurements were performed on days 1–17, in the morning 30 min after morphine and/or PEA acute administration.

### Paw-Pressure Test

The nociceptive threshold of rats was determined with an analgesimeter (Ugo Basile, Varese, Italy), according to the method described by Leighton et al. (1988). A constantly increasing pressure was applied to a small area of the dorsal surface of the hind paw using a blunt conical probe by a mechanical device. While rats were lightly restrained, mechanical pressure was increased until vocalization or a withdrawal reflex occurred. Vocalization or withdrawal reflex thresholds were expressed in grams. Rats scoring below 40 g or over 75 g during the test before drug administration were rejected (25%). For analgesia measurements, mechanical pressure application was stopped at 200 g.

### Irwin Test

Each rat was individually placed in a transparent cage (26 cm × 41 cm), and 26 neurobehavioral or physiological parameters were systematically assessed according to Irwin (1968). Behavioral, autonomic, and neurological manifestations produced by compound administration in rats were evaluated: motor displacement, motor reflexes, stereotypies, grooming, reaction to painful or environmental stimuli (analgesia, irritability), startle response, secretions, excretions, respiratory movements, skin color and temperature, piloerection, exophthalmos, eyelid and corneal reflexes, muscle tone, ataxia, tremors, head twitches, jumps, convulsions, Straub tail, and other signs or symptoms. For postural reflexes (righting reflex) and other signs such as piloerection, exophthalmia (exaggerated protrusion of the eyeball), ataxia, tremors, and Straub tail, only presence or absence was recorded. Skin color was evaluated qualitatively (pale, red, or purple); other signs were evaluated semi-quantitatively, according to the observer’s personal scale (0 to +4, -4 to 0, or -4 to +4). The terms sedation and excitation express the final interpretation of a group of signs: reduced motor activity, reduced startle response, eyelid ptosis, and reduced response to manual manipulation with regard to the former; and increased motor activity, increased startle response, increased response to manual manipulation, and exophthalmia with regard to the latter. Hyperactivity included running, jumps, and attempts to escape from the container.

### Statistical Analysis

Trained observers, not informed about the specific treatment of each animal group carried out the tests. Results were expressed as means ± SEM and analysis of variance was performed by ANOVA test. A Bonferroni’s significant difference procedure was used as a *post hoc* comparison. *P*-values less than 0.05 were considered significant. Data were analyzed using “Origin 8.1” software.

## Results and Discussion

The capacity of PEA to modulate the protective responses against different damages to the nervous tissue proposes this endogenous molecule as a component of the cellular homeostatic system (Skaper et al., 2013). In some pathological conditions, PEA endogenous production seems to be inadequate leading to the need for exogenous supplementation. In order to favor the homeostatic processes mediated by PEA, we decided to explore the effect of PEA pretreatment on morphine analgesia and tolerance development. Rats were treated with PEA (1–30 mg/kg, p.o.) daily (in the evening) from day -8 for the duration of the experiment (day 12). On day 1 (16 h after the last PEA administration), morphine (10 mg/kg s.c.) was injected. Thirty minutes later, the pain threshold was measured by Paw pressure test. The pretreatment with PEA dose-dependently increased the responsiveness to morphine (**Figure 1**): animals tolerated 171.7 ± 14.2 g (30 mg/kg PEA + morphine) on the posterior paw in comparison to 128.6 ± 5.2 g (vehicle + morphine) starting from a basal value of 67.2 ± 0.3 g (vehicle + vehicle). In the following days, morphine efficacy progressively decreased. On day 7, vehicle + morphine-treated animals completely lacked the analgesic response whereas rats pretreated with PEA and morphine exhibited a delay in the onset of tolerance till day 12 (**Figure 1**). Interestingly, the extension of morphine response was promoted by PEA also in the absence of the pretreatment (as previously published, Di Cesare Mannelli et al., 2015a), instead pretreatment is necessary to increase morphine efficacy in the first days. On the other hand, the modulatory role mediated by PEA on mast and glial cells as well as on receptor signaling [peroxisome proliferator-activated receptor (PPAR)-α; cannabinoid; Di Cesare Mannelli et al., 2015b] can promote phenomena of cellular plasticity able to enhance morphine analgesia after a repeated treatment in naïve animals. The same groups were monitored over time to assess the recovery from tolerance measuring the responsiveness to morphine. During the recovery period, PEA-treated animals continued to receive the compound daily (10 and 30 mg/kg) whereas control groups were treated with vehicle. Morphine (10 mg/kg) was injected on selected days. As shown in **Figure 2**, 4 days *post* tolerance development (d.p.t., tolerance was considered as the complete lack of morphine effect, separately for each group), PEA-treated groups had already regained sensitivity to morphine (Paw pressure test, 30 min after injection). At least for the higher dosed group (30 mg/kg PEA), the antinociceptive effect was registered also on 10, 14, 21, 31 d.p.t. The lower dose did not seem to be able to sustain a stable effect. Control rats treated with vehicle exhibited recovery from tolerance 31 d.p.t. only (**Figure 2**, vehicle + morphine). After recovery of tolerance, no treatment group reached the full analgesic effects obtained on day 1. Nevertheless, PEA-treated animals showed antinociception as intense as morphine on day 3 before the onset of tolerance (**Figure 2**, dashed line).

As previously published, PEA counteracts pain hypersensitivity without modifying the normal nociception (Luongo et al., 2013; Di Cesare Mannelli et al., 2015b). Conditions of hypersensitivity, like neuropathic pain, share common characteristics with opioid tolerance. Among others, the plasticity of glial cells is present in both conditions (Mika, 2008; Di Cesare Mannelli et al., 2013b, 2014) whereas glial inhibitors like minocycline or fluorocitrate attenuate both morphine tolerance and neuropathic pain (Mika et al., 2009; Di Cesare Mannelli et al., 2014). PEA is a modulator of the glial response (Skaper et al., 2013; Di Cesare Mannelli et al., 2015a) and is active against neuropathic pain (Di Cesare Mannelli et al., 2013a) so we were excited to evaluate the effect of PEA in morphine tolerant rats. Different groups were treated daily (in the evening) p.o. with PEA (30 mg/kg) or vehicle; morphine (10 mg/kg s.c.) was injected from day 1 till the complete loss of antinociceptive response was reached. When tolerance was well-established (4 d.p.t. for each group), the pain threshold (Paw pressure test) was measured in the morning before (0 min) and 30 min after an acute treatment with PEA (30 mg/kg, p.o.). **Figure 3** shows the expected lack of antinociceptive effect of PEA in control (vehicle + vehicle) as well as in the morphine-tolerant rats pretreated with vehicle. On the contrary, the morphine-tolerant rats pretreated with PEA exhibited antinociception upon its acute administration, as evidenced by an increase in pain threshold up to 86.3 ± 4.1 g (30 min; in comparison to 64.9 ± 1.2 g, pretest value at 0 min).

On the basis of this evidence, we decided to set up a preclinical protocol focused on maintaining of a stable antinociception based on the use of preemptive PEA followed by the acute association of morphine and PEA. The purpose was to obtain daily, over time, the same increase of pain threshold (arbitrarily fixed at 90 ± 10 g, as measured by Paw pressure, starting from a basal value of about 65 ± 5 g) using the lowest possible morphine dosages. Rats were treated with vehicle (group a) or PEA (30 mg/kg; groups b, c, and d) p.o. daily (in the evening) for the duration of the experiment starting on day -8. From day 1 till day 17, daily pain measurements were performed in the morning (16 h after the last preemptive PEA administration), before and 30 min after an acute treatment with increasing doses of morphine (5–100 mg/kg, s.c., groups a and b) or different combinations of morphine (5–20 mg/kg, s.c.) and PEA (30–120 mg/kg, p.o.) (groups c and d). Day by day results are shown in the **Supplementary Figures S1A–D**. **Figure 4** shows the results of representative days. On day 1, 5 mg/kg morphine s.c. increased the pain threshold of group a to 87.2 ± 1.1 g. As expected, the other groups pretreated with PEA showed higher responses to morphine, reaching about 110 g. On day 4, 10 mg/kg morphine was needed to maintain antinociception up to about 90 g (group a) whereas 5 mg/kg were enough for group b. The combination of 5 mg/kg morphine and 30 mg/kg PEA boosted the increase up to about 110 g. On day -8, 16 mg/kg morphine was active in group a, 10 mg/kg induced higher effects in groups b and c (preemptive PEA), and only 7 mg/kg morphine were necessary when combined with 30 mg/kg PEA. On day 12, the acute administration of 7 mg/kg morphine associated with 90 mg/kg PEA (group d) evoked a better effect than 20 mg/kg morphine alone (group a). Interestingly, as further described in **Figure 3**, also the acute administration of 30 mg/kg PEA alone induced a modest but significant effect (group c). On day 17, a dramatic increase of morphine (100 mg/kg) was needed to induce a significant effect in groups a and b, and PEA association allowed efficacy using 20 mg/kg morphine/90 mg/kg PEA or 13 mg/kg morphine/120 mg/kg PEA in groups c and d, respectively. The same tests were conducted on a group pre- (30 mg/kg) and acutely- (30–120 mg/kg) treated with PEA alone (group e). As shown in **Supplementary Table S1**, the pain threshold of these animals was not modified by administration of PEA. Moreover, the pain threshold of each groups (a–e) was not significantly different at the pretest performed every day (0 min) before acute administration (**Supplementary Table S2**). Similar values (about 65 g) were shown by the control group that received vehicle + vehicle treatments (group f; data not shown).

**Figure 5** summarizes the results showing the trend of dosage increase (days 1–17) to obtain a significant antinociception (90 ± 10 g) in group a (preemptive vehicle + acute morphine + acute vehicle) in comparison with groups c and d (preemptive PEA + acute morphine + acute PEA). The doses of acutely administered morphine and PEA are reported.

Finally, the good tolerability of PEA is described in several clinical studies (Kahlich et al., 1979; Mazzari et al., 1996; Keppel Hesselink et al., 2013). Accordingly, the present data showed that the repeated treatment with increasing doses of PEA (group e) did not induce behavioral, autonomic or neurological alteration as evaluated by the Irwin test (**Supplementary Table S3**).

Our results agree with a modulatory role of the natural lipid PEA in the nervous system. The pharmacodynamic involves receptor-mediated signals as the activation of the α-subtype (and, lesser, the δ and γ subtypes; Paterniti et al., 2013) of the peroxisome proliferator-activated receptors able to evoke pain relief and neurorestoration (LoVerme et al., 2006; Di Cesare Mannelli et al., 2013a), and endocannabinoid-driven actions (on the basis of a PEA-mediated enhancement of endogenous compounds by potentiating their affinity for receptors or by inhibiting their metabolic degradation; Calignano et al., 1998). Moreover, PEA increases the antioxidant defense (Mattace Raso et al., 2013) and exerts antiinflammatory effects (enhancement of IκB-α, decrease of COX-2 in the spinal cord; Di Cesare Mannelli et al., 2015b), counteracting two characteristic features of morphine tolerance development (Abdel-Zaher et al., 2013; Eidson et al., 2017). Redox imbalance (Abdel-Zaher et al., 2013) and inflammation (Eidson et al., 2017) decrease the analgesic effects of morphine whereas endocannabinoids (Wilkerson et al., 2017) and PPARs (at least for the γ-subtype; de Guglielmo et al., 2014) modulations are positive regulators of morphine antinociception.

Moreover, acute and chronic morphine evokes a CNS glial cell response that actively opposes the analgesic effects of morphine and contributes to the development of tolerance (Eidson and Murphy, 2013). Repeated morphine treatments activate microglia and astrocyte cells, enhancing density and favoring cell hypertrophy. On the contrary, administration of pharmacological glial inhibitors attenuates tolerance to morphine analgesia (Song and Zhao, 2001; Mika, 2008; Mika et al., 2009). Interestingly, PEA seems to be able to modulate glial cells instead to act as a general depressor of glial functions (Skaper et al., 2013). The homeostatic properties of PEA may allow the inhibition of glial hyper-reactivity, thus PEA prevents microglia and astrocyte activation in models of inflammatory and neuropathic pain (Esposito et al., 2011; Luongo et al., 2013; Di Cesare Mannelli et al., 2015b). Recently, we demonstrated the property of PEA of attenuating morphine tolerance by a glia-mediated mechanism (Di Cesare Mannelli et al., 2015a). Interestingly, the Nobel prize winner Rita Levi-Montalcini suggested PEA as an autacoid negatively modulating the behavior of mast cells in response to noxious stimuli [autacoid local inflammation antagonism (ALIA); Aloe et al., 1993]. PEA reduces mast cell degranulation controlling CNS damages by limiting mast cell infiltration and activation (Skaper et al., 2013). The increased number of mast cells could increase the blood brain barrier (BBB) permeability and activate microglia, astrocytes, and T cells (Kempuraj et al., 2017). Mast cells are resident in the CNS (Skaper et al., 2015), and are also able to cross the BBB into the brain from the peripheral tissue in neuroinflammatory conditions (Florenzano and Bentivoglio, 2000; Skaper et al., 2012; Silver and Curley, 2013) as well as in physiological conditions (Silverman et al., 2000). The key mast cell mediator, histamine, has sensitizing effects on nociceptors, as well as other soluble factors secreted by mastocytes. Mast cell degranulation is a principal source of rapid release of proalgic nerve growth factor (NGF), and mast cells respond in a paracrine/autocrine fashion to NGF (Skaper et al., 2013). The capacity of PEA to modulate protective responses during inflammation and pain led to the hypothesis that endogenous PEA may be a component of the complex homeostatic system controlling the basal threshold of pain. In 2014, opioids were involved in about 30000 deaths in the United States, mostly related to natural and semisynthetic opioids, a tripling of the rate of opioid overdoses compared to 2000 (Rudd et al., 2016a,b; Manchikanti et al., 2017). Data from the Centers for Disease Control and Prevention show that the United States’ opioid overdose epidemic includes an increase in overdose deaths involving prescription opioid pain relievers (Rudd et al., 2016a,b). The problem is mainly related to treatment of persistent pain based on increasing doses of opioids. Systematic assessment of evidence showed that opioid doses of 200 morphine milligram equivalents (MME) per day increased mortality rates gradually, higher than the doses of 100 MME or more per day, increasing the risks for opioid overdose by factors of 2.0 to 8.9 (Dasgupta et al., 2016; Manchikanti et al., 2017).

The present results suggest PEA as a powerful enhancer of morphine antinociception able to allow maintenance of low dosages and long-lasting activity, attenuating tolerance development. This preclinical approach is proposed for a clinical validation facilitated by PEA safety profile.

## Author Contributions

LM and EL performed the *in vivo* experiments. CG and LDCM conceived the study, planned its design, and drafted the manuscript.

## Conflict of Interest Statement

CG and LDCM received a grant from Epitech (Padova, Italy).The other authors declare that the research was conducted in the absence of any commercial or financial relationships that could be construed as a potential conflict of interest.

## Supplementary Material

FIGURE S1 **(A,B)** Induction of antinociception over time with different combinations of morphine and PEA. Rats were treated with vehicle (group a) or PEA (30 mg/kg; groups b, c, and d) p.o. daily (in the evening) for the duration of the experiment starting on day -8. To maintain a significant increase of pain threshold (90 ± 10 g) vs. baseline (control; vehicle + vehicle + vehicle, not shown), beginning on day 1, increasing daily doses of morphine (5–100 mg/kg) were injected s.c. to groups a and b. Measurements were performed on days 1–17, in the morning 30 min after morphine and/or PEA acute administration. Data are expressed as the mean ± SEM of values from 12 rats analyzed in two different experimental sets. **(C,D)** Rats were treated with PEA (30 mg/kg; groups c and d) p.o. daily (in the evening) for the duration of the experiment starting on day -8. To maintain a significant increase of pain threshold (90 ± 10 g) vs. baseline (control; vehicle + vehicle + vehicle, not shown), beginning on day 1, different daily combinations of morphine (5–100 mg/kg, s.c.) and PEA (30–120 mg/kg, p.o.) were administered to groups c and d. Measurements were performed on days 1–17, in the morning 30 min after morphine or/and PEA acute administration. Data are expressed as mean ± SEM of values from 12 rats analyzed in two different experimental sets.

TABLE S1 Effect of repeated treatments with PEA on the pain threshold measured by the Paw pressure test.

TABLE S2 Induction of anti-nociception over time with different combination of morphine and PEA Paw pressure test.

TABLE S3 Effect of repeated treatments with PEA on behavioral, autonomic, and neurological manifestations by the Irwin test.
