# Supplementary figures and images for: Ultramicronized N-Palmitoylethanolamine Supplementation for Long-Lasting, Low-Dosed Morphine Antinociception
Source: Front Pharmacol. 2018 Jun 1;9:473. doi: 10.3389/fphar.2018.00473 (PMC5992817; doi:10.3389/fphar.2018.00473)

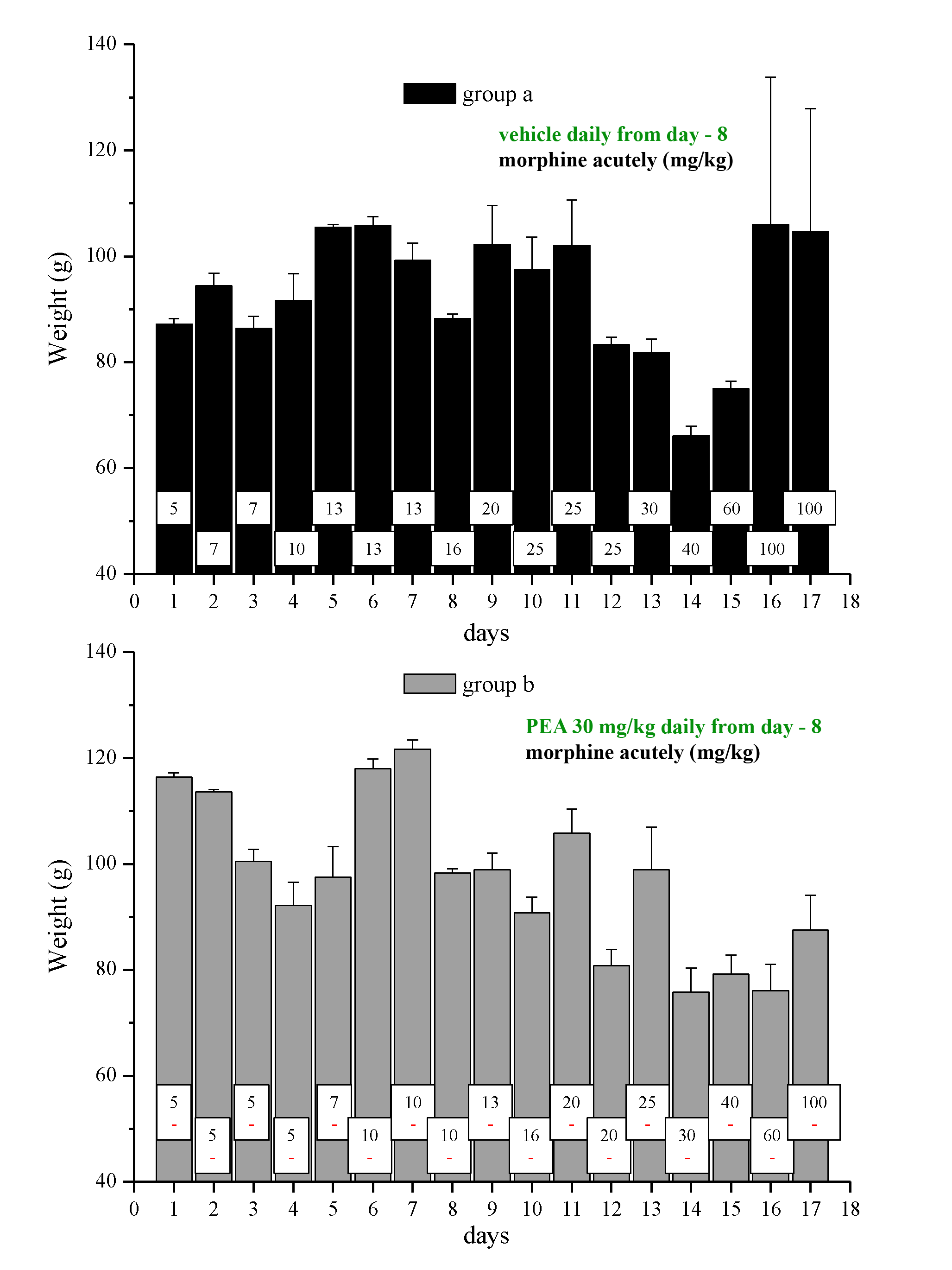

Supplement: FIGURE S1 — (A,B) Induction of antinociception over time with different combinations of morphine and PEA. Rats were treated with vehicle (group a) or PEA (30 mg/kg; groups b, c, and d) p.o. daily (in the evening) for the duration of the experiment starting on day -8. To maintain a significant increase of pain threshold (90 ± 10 g) vs. baseline (control; vehicle + vehicle + vehicle, not shown), beginning on day 1, increasing daily doses of morphine (5–100 mg/kg) were injected s.c. to groups a and b. Measurements were performed on days 1–17, in the morning 30 min after morphine and/or PEA acute administration. Data are expressed as the mean ± SEM of values from 12 rats analyzed in two different experimental sets. (C,D) Rats were treated with PEA (30 mg/kg; groups c and d) p.o. daily (in the evening) for the duration of the experiment starting on day -8. To maintain a significant increase of pain threshold (90 ± 10 g) vs. baseline (control; vehicle + vehicle + vehicle, not shown), beginning on day 1, different daily combinations of morphine (5–100 mg/kg, s.c.) and PEA (30–120 mg/kg, p.o.) were administered to groups c and d. Measurements were performed on days 1–17, in the morning 30 min after morphine or/and PEA acute administration. Data are expressed as mean ± SEM of values from 12 rats analyzed in two different experimental sets. [file Image_1.TIF]

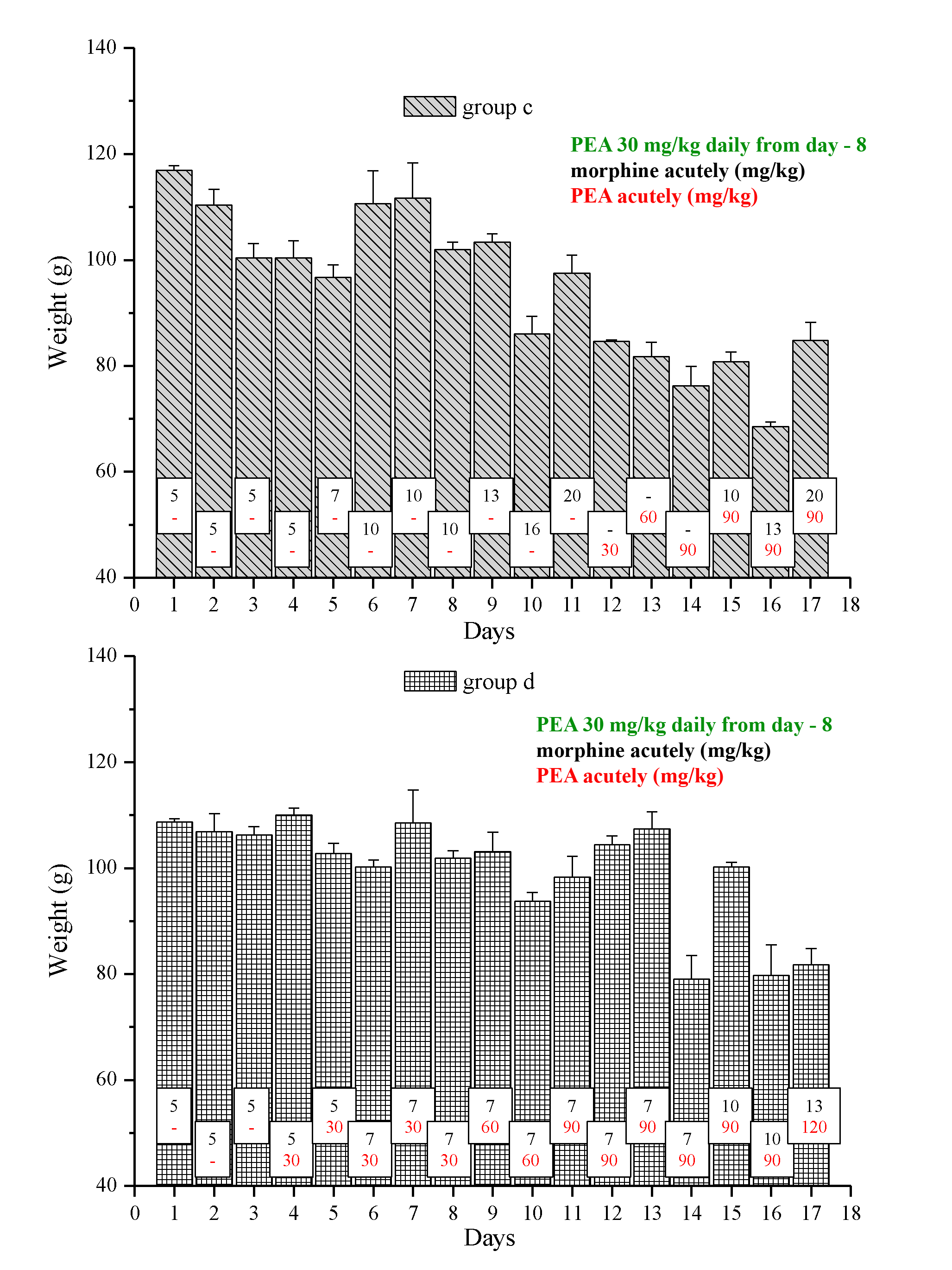

Supplement: Supplementary file 2 [file Image_2.TIF]
