# Supplementary material for: Ultramicronized N-Palmitoylethanolamine Supplementation for Long-Lasting, Low-Dosed Morphine Antinociception
Source: Front Pharmacol. 2018 Jun 1;9:473. doi: 10.3389/fphar.2018.00473 (PMC5992817; doi:10.3389/fphar.2018.00473)
Supplement: TABLE S1 — Effect of repeated treatments with PEA on the pain threshold measured by the Paw pressure test. [file Table_1.doc]

| **Supplementary Table S1**  Effect of repeated treatments with PEA on the pain threshold measured by the Paw pressure test | | | | |
| --- | --- | --- | --- | --- |
|  | | | **Weight (g)** | |
|  |  | *Treatment* | *Time (min)* | |
| day | group | **PEA**  *(mg/kg p.o.)* | 0 min | 30 min |
| -9 | e | - | 65.3 ± 0.9 | - |
| from -8 to 0 | e | 30 | - | - |
| 1 | e | 30 | 64.2 ± 1.2 | 61.9 ± 2.6 |
| 2 | e | 30 | 66.3 ± 0.9 | 62.7 ± 1.4 |
| 3 | e | 30 | 64.4 ± 0.6 | 66.1 ± 2.0 |
| 4 | e | 30 *+* 30 acute | 65.5 ± 1.5 | 64.7 ± 1.2 |
| 5 | e | 30 *+* 30 acute | 62.8 ± 1.5 | 65.8 ± 1.7 |
| 6 | e | 30 *+* 30 acute | 64.7 ± 1.1 | 63.7 ± 0.5 |
| 7 | e | 30 *+* 30 acute | 65.0 ± 1.7 | 67.1 ± 2.1 |
| 8 | e | 30 *+* 30 acute | 62.9 ± 3.1 | 64.2 ± 0.8 |
| 9 | e | 30 *+* 60 acute | 63.1 ± 1.2 | 66.8 ± 0.7 |
| 10 | e | 30 *+* 60 acute | 67.2 ± 0.3 | 65.0 ± 1.0 |
| 11 | e | 30 *+* 90 acute | 66.4 ± 0.7 | 66.1 ± 2.4 |
| 12 | e | 30 *+* 90 acute | 66.7 ± 2.2 | 67.5 ± 1.4 |
| 13 | e | 30 *+* 90 acute | 65.4 ± 1.0 | 62.0 ± 0.9 |
| 14 | e | 30 *+* 90 acute | 61.3 ± 1.5 | 68.4 ± 1.6 |
| 15 | e | 30 *+* 90 acute | 63.6 ± 1.4 | 65.8 ± 0.9 |
| 16 | e | 30 *+* 90 acute | 65.0 ± 1.0 | 66.4 ± 1.6 |
| 17 | e | 30 *+* 120 acute | 63.6 ± 0.7 | 65.5 ± 1.5 |

Rats were treated with PEA (30 mg/kg; group e) p.o. daily (in the evening) for the duration of the experiment starting on day -8. Pain threshold was evaluated by the Paw pressure test every day (1 - 17) in the morning. Measurements were performed before and, when specified, 30 min after PEA acute administration. Data are expressed as the mean ± S.E.M. of values from 12 rats analyzed in 2 different experimental sets.
