# Supplementary material for: Ultramicronized N-Palmitoylethanolamine Supplementation for Long-Lasting, Low-Dosed Morphine Antinociception
Source: Front Pharmacol. 2018 Jun 1;9:473. doi: 10.3389/fphar.2018.00473 (PMC5992817; doi:10.3389/fphar.2018.00473)
Supplement: TABLE S2 — Induction of anti-nociception over time with different combination of morphine and PEA Paw pressure test. [file Table_2.doc]

| **Supplementary Table S2**  Induction of anti-nociception over time with different combination of morphine and PEA  Paw pressure test | | | | | |
| --- | --- | --- | --- | --- | --- |
|  |  | *Treatments* | | *Time (min)* | |
| day | group | **morphine**  *(mg/kg s.c.)* | **PEA**  *(mg/kg p.o.)* | 0 min | 30 min |
| 1 | a | 5 | - | 65.5 ± 1.1 | 87.2 ± 1.1** |
|  | b | 5 | 30 | 63.3 ± 0.8 | 116.4 ± 0.8**°° |
|  | c | 5 | 30 | 62.5 ± 0.9 | 116.9 ± 0.9**°° |
|  | d | 5 | 30 | 65.8 ± 1.4 | 108.7 ± 0.6**°° |
| 2 | a | 7 | - | 64.7 ± 1.2 | 94.4 ± 2.4** |
|  | b | 5 | 30 | 67.5 ± 0.5 | 113.6 ± 0.5**°° |
|  | c | 5 | 30 | 64.6 ± 1.5 | 110.4 ± 2.9**°° |
|  | d | 5 | 30 | 64.8 ± 1.1 | 106.9 ± 3.4**° |
| 3 | a | 7 | - | 66.1 ± 1.5 | 86.4 ± 2.2** |
|  | b | 5 | 30 | 63.8 ± 1.5 | 100.5 ± 2.2**°° |
|  | c | 5 | 30 | 63.9 ± 0.9 | 100.4 ± 2.7**°° |
|  | d | 5 | 30 | 63.7 ± 1.3 | 106.3 ± 1.6**°° |
| 4 | a | 10 | - | 62.5 ± 1.4 | 91.6 ± 5.1** |
|  | b | 5 | 30 | 61.7 ± 0.8 | 92.2 ± 4.3** |
|  | c | 7 | 30 | 62.1 ± 0.7 | 100.4 ± 3.2** |
|  | d | 5 | 30 *+* 30*co-ad.* | 62.9 ± 0.8 | 110.0 ± 1.3**° |
| 5 | a | 13 | - | 62.2 ± 1.5 | 105.5 ± 0.5** |
|  | b | 7 | 30 | 62.8 ± 1.5 | 97.5 ± 5.8** |
|  | c | 7 | 30 | 62.1 ± 0.8 | 96.7 ± 2.4** |
|  | d | 5 | 30 *+* 30*co-ad.* | 63.1 ± 0.7 | 102.8 ± 1.9** |
| 6 | a | 13 | - | 63.0 ± 1.9 | 105.8 ± 1.7** |
|  | b | 10 | 30 | 63.0 ± 1.0 | 118.0 ± 1.9** |
|  | c | 10 | 30 | 64.2 ± 1.4 | 110.6 ± 6.2** |
|  | d | 7 | 30 *+* 30*co-ad.* | 62.9 ± 1.6 | 100.2 ± 1.3** |
| 7 | a | 13 | - | 64.4 ± 1.5 | 99.2 ± 3.3** |
|  | b | 10 | 30 | 61.9 ± 1.0 | 121.7 ± 1.7**°° |
|  | c | 10 | 30 | 62.1 ± 0.3 | 111.7 ± 6.6** |
|  | d | 7 | 30 *+* 30*co-ad.* | 64.8 ± 1.6 | 108.5 ± 6.2** |
| 8 | a | 16 | - | 64.7 ± 1.2 | 88.3 ± 0.8** |
|  | b | 10 | 30 | 66.4 ± 0.7 | 98.3 ± 0.8**°° |
|  | c | 10 | 30 | 66.2 ± 1.6 | 101.9 ± 1.4**°° |
|  | d | 7 | 30 *+* 30*co-ad.* | 63.1 ± 0.7 | 103.1 ± 1.2**°° |
| 9 | a | 20 | - | 62.2 ± 1.5 | 102.2 ± 7.4** |
|  | b | 13 | 30 | 63.6 ± 0.7 | 98.9 ± 3.1** |
|  | c | 13 | 30 | 67.5 ± 2.1 | 103.3 ± 1.6** |
|  | d | 7 | 30 *+* 60*co-ad.* | 61.2 ± 0.8 | 103.1 ± 3.7** |
| 10 | a | 25 | - | 63.3 ± 0.5 | 97.5 ± 6.1** |
|  | b | 16 | 30 | 64.7 ± 1.7 | 90.8 ± 2.9** |
|  | c | 16 | 30 | 63.8 ± 1.3 | 86.0 ± 3.4** |
|  | d | 7 | 30 *+* 60*co-ad.* | 64.6 ± 0.8 | 93.8 ± 1.6** |
| 11 | a | 25 | - | 61.7 ± 0.8 | 102.1 ± 8.5** |
|  | b | 20 | 30 | 67.2 ± 1.5 | 105.8 ± 4.6** |
|  | c | 20 | 30 | 65.4 ± 1.5 | 97.5 ± 3.4** |
|  | d | 7 | 30 *+* 90*co-ad.* | 65.6 ± 0.6 | 98.3 ± 3.9** |
| 12 | a | 25 | - | 60.8 ± 0.8 | 83.3 ± 1.4** |
|  | b | 20 | 30 | 65.0 ± 1.0 | 80.8 ± 3.0** |
|  | c | - | 30 + 30*acute* | 65.2 ± 1.3 | 84.6 ± 3.3** |
|  | d | 7 | 30 *+* 90*co-ad.* | 65.2 ± 1.3 | 104.4 ± 1.7**°° |
|  |  | *Treatments* | | *Time (min)* | |
| day | group | **morphine**  *(mg/kg s.c.)* | **PEA**  *(mg/kg p.o.)* | 0 min | 30 min |
| 13 | a | 30 | - | 61.7 ± 0.8 | 81.7 ± 2.7** |
|  | b | 25 | 30 | 64.4 ± 0.6 | 98.9 ± 8.1** |
|  | c | - | 30 + 60*acute* | 65.0 ± 1.0 | 86.4 ± 4.4** |
|  | d | 7 | 30 *+* 90*co-ad.* | 66.2 ± 1.0 | 107.5 ± 3.2**°° |
| 14 | a | 40 | - | 61.1 ± 0.5 | 66.1 ± 1.8 |
|  | b | 30 | 30 | 65.5 ± 0.5 | 75.8 ± 4.6 |
|  | c | - | 30 + 90*acute* | 61.5 ± 2.1 | 76.2 ± 3.7* |
|  | d | 7 | 30 *+* 90*co-ad.* | 63.5 ± 1.5 | 79.0 ± 4.5* |
| 15 | a | 60 | - | 66.4 ± 0.7 | 75.0 ± 1.4* |
|  | b | 40 | 30 | 64.1 ± 1.8 | 79.2 ± 3.6* |
|  | c | 10 | 30 + 90*co-ad.* | 64.8 ± 1.1 | 80.8 ± 1.8** |
|  | d | 10 | 30 *+* 90*co-ad.* | 62.9 ± 0.2 | 100.2 ± 0.9**°° |
| 16 | a | 100 | - | 64.4 ± 1.5 | 172.2 ± 27.8* |
|  | b | 60 | 30 | 61.1 ± 1.1 | 76.1 ± 4.9 |
|  | c | 13 | 30 + 90*co-ad.* | 64.3 ± 0.9 | 68.5 ± 0.9 |
|  | d | 10 | 30 *+* 90*co-ad.* | 63.1 ± 0.7 | 79.8 ± 5.7 |
| 17 | a | 100 | - | 62.7 ± 2.0 | 106.7 ± 23.1 |
|  | b | 100 | 30 | 57.8 ± 1.5 | 87.5 ± 6.6* |
|  | c | 20 | 30 + 90*co-ad.* | 57.1 ± 1.6 | 84.8 ± 3.4** |
|  | d | 13 | 30 *+* 120*co-ad.* | 60.6 ± 1.0 | 81.8 ± 3.0** |

Morphine was dissolved in saline solution and daily subcutaneously (s.c.) administered. Pea was suspended in 1% CMC and daily *per os* (p.o.) administered. Treatment with PEA started 8 days before the first morphine injection and continued during all the experiment. Paw pressure test was performed before (0 min) and after (30 min) morphine and/or Pea administration.

**Treatment’s group:**

1. morphine + vehicle (the dose of morphine was increased during days)
2. morphine + PEA (the dose of morphine and Pea were increased during days)
3. morphine + PEA (the dose of morphine and Pea were increased during days. From day 12 to day 14, morphine injection was stopped and analgesia was measured 30 min after Pea administration. From day 15, animals received two administration of PEA, one in the evening and one in co-administration with morphine)
4. morphine + PEA (the doses of morphine and PEA were increased during days. From day 4, animals received two administration of PEA, one in the evening and one in co-administration with morphine)

Data are expressed as the mean ± S.E.M. of values from 12 rats analyzed in 2 different experimental sets. *P<0.05 and **P<0.01 *vs* 0 min of the same group; °P<0.05 and °°P<0.01 vs 30 min of group a.
