# Supplementary material for: Ultramicronized N-Palmitoylethanolamine Supplementation for Long-Lasting, Low-Dosed Morphine Antinociception
Source: Front Pharmacol. 2018 Jun 1;9:473. doi: 10.3389/fphar.2018.00473 (PMC5992817; doi:10.3389/fphar.2018.00473)
Supplement: TABLE S3 — Effect of repeated treatments with PEA on behavioral, autonomic, and neurological manifestations by the Irwin test. [file Table_3.DOC]

| **Supplementary Table S3**  Effect of repeated treatments with PEA on behavioural, autonomic, and neurological manifestations  by the Irwin test | | | |
| --- | --- | --- | --- |
|  | Group e (PEA treatment) | | Limits |
|  | Day -8 | Day 17 |  |
| **Behaviour** |  |  |  |
| *Spontaneous activity* | 4 | 4 | 4-0 |
| *Passivity* | 0 | 0 | 0-4 |
| *Cleaning* | 4 | 4 | 4-0 |
| *Curiosity* | 4 | 4 | 4-0 |
| *Reactivity* | 4 | 4 | 4-0 |
| *Vocalization* | 0 | 0 | 0-4 |
| **S.N.C. excitement** |  |  |  |
| *Straub tail* | 0 | 0 | 0-4 |
| *Tremors* | 0 | 0 | 0-4 |
| *Convulsions* | 0 | 0 | 4-0 |
| **Movement** |  |  |  |
| *Ataxia* | 0 | 0 | 0-4 |
| *Stereotipies* | 0 | 0 | 0-4 |
| *Straightening reflex* | 4 | 4 | 4-0 |
| **Muscolar tone** |  |  |  |
| *Physical strenght* | 4 | 4 | 4-0 |
| **Reflexes** |  |  |  |
| *Palpebral reflex* | 4 | 4 | 4-0 |
| **Autonomic signes** |  |  |  |
| *Piloerection* | 0 | 0 | 0-4 |
| *Exolphthalmos* | 0 | 0 | 0-4 |
| *Cyanosis* | 0 | 0 | 0-4 |
| *Flush* | 0 | 0 | 0-4 |
| *Pallor* | 0 | 0 | 0-4 |
| *Palpebral opening* | 4 | 4 | 4-0 |
| *Salivation* | 0 | 0 | 0-4 |
| *Lacrimation* | 0 | 0 | 0-4 |
| *Hypo-hyperthermia* | 0 | 0 | -4/+4 |
| *Writhing* | 0 | 0 | 0-4 |
| **Toxicity** |  |  |  |
| *Immediate death* | 0 | 0 | 0-4 |
| *Delayed death (48 h)* | 0 | 0 | 0-4 |

Rats were treated with PEA (30 mg/kg; group e) p.o. daily (in the evening) for the duration of the experiment starting on day -8. Adjunctive acute treatment with PEA were performed as specified in the Supplementary Table S1. The Irwin test was performed before treatments on day -8 and at the end of the experiments on day 17, 30 min after the acute administration of 120 mg/kg PEA p.o.. Data are from 12 rats analyzed in 2 different experimental sets.
